# 1,3-Bis(2,6-diisopropyl­phen­yl)imidazolidinium tetra­phenyl­borate dichloro­methane disolvate

**DOI:** 10.1107/S1600536810028424

**Published:** 2010-07-24

**Authors:** Nick A. Giffin, Arthur D. Hendsbee, Jason D. Masuda

**Affiliations:** a Department of Chemistry, Saint Mary’s University, 923 Robie Street, Halifax, NS, Canada B3H 3C3

## Abstract

The title compound, C_27_H_39_N_2_^+^·C_24_H_20_B^−^·2CH_2_Cl_2_, is the first reported imidazolidinium cation with the sterically demanding 2,6-diisopropyl­phenyl groups in the 1,3-positions. The crystal structure is stabilized by weak inter­molecular C—H⋯π(arene) inter­actions. Due to the bulky nature of both the flanking 2,6-diisopropyl­phenyl substituents and the tetra­phenyl­borate counter-ion, anion inter­actions with the imidazolidinium H atom in the 2-position are not observed, also a first for this class of *ortho*-alkyl-substituted Arduengo-type carbene precursors.

## Related literature

There are few examples in the literature of crystallographically characterized imidazolium or imidazolidinium complexes with *ortho*-alkyl substituted phenyl groups in the 1,3-positions, see: Arduengo *et al.* (1995 ▶, 1999 ▶); Fliedel *et al.* (2007 ▶); Hagos *et al.* (2008 ▶).
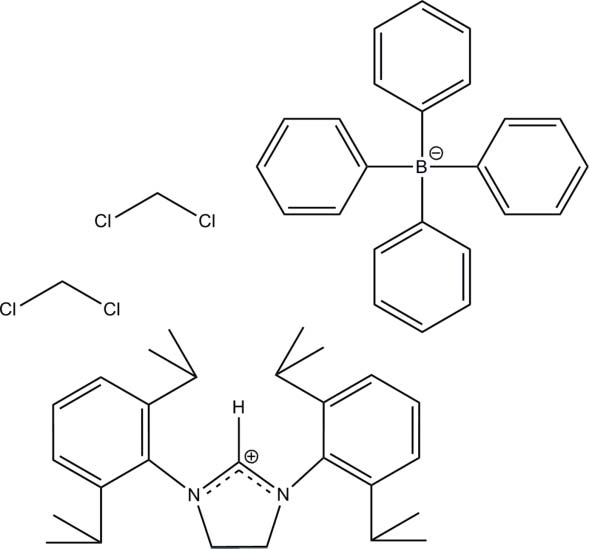


## Experimental

### 

#### Crystal data

C_27_H_39_N_2_^+^·C_24_H_20_B^−^·2CH_2_Cl_2_
*M* *_r_* = 880.66
Monoclinic, 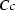

*a* = 21.4648 (14) Å
*b* = 10.3964 (7) Å
*c* = 22.7524 (15) Å
β = 93.760 (1)°
*V* = 5066.4 (6) Å^3^
*Z* = 4
Mo *K*α radiation
μ = 0.27 mm^−1^
*T* = 296 K
0.49 × 0.34 × 0.29 mm

#### Data collection

Bruker APEXII CCD diffractometer
Absorption correction: multi-scan (*SADABS*; Bruker, 2010 ▶) *T*_min_ = 0.675, *T*_max_ = 0.746
17851 measured reflections
8396 independent reflections
7118 reflections with *I* > 2σ(*I*)
*R*_int_ = 0.013

#### Refinement

*R*[*F* ^2^ > 2σ(*F* ^2^)] = 0.062
*wR*(*F* ^2^) = 0.186
*S* = 1.35
8396 reflections
549 parameters
892 restraints
H-atom parameters constrained
Δρ_max_ = 0.41 e Å^−3^
Δρ_min_ = −0.44 e Å^−3^
Absolute structure: Flack (1983 ▶), 3456 Friedels
Flack parameter: −0.11 (10)

### 

Data collection: *APEX2* (Bruker, 2010 ▶); cell refinement: *SAINT* (Bruker, 2010 ▶); data reduction: *SAINT*; program(s) used to solve structure: *SHELXS97* (Sheldrick, 2008 ▶); program(s) used to refine structure: *SHELXL97* (Sheldrick, 2008 ▶); molecular graphics: *ORTEP-3 for Windows* (Farrugia, 1997 ▶); software used to prepare material for publication: *SHELXTL* (Sheldrick, 2008 ▶).

## Supplementary Material

Crystal structure: contains datablocks I, global. DOI: 10.1107/S1600536810028424/lh5077sup1.cif

Structure factors: contains datablocks I. DOI: 10.1107/S1600536810028424/lh5077Isup2.hkl

Additional supplementary materials:  crystallographic information; 3D view; checkCIF report

## Figures and Tables

**Table 1:** Hydrogen-bond geometry (Å, °) *Cg*1, *Cg*2, *Cg*3 and *Cg*4 are the centroids defined by the ring atoms C28–C33, C34–C39, C40–C45 and C46–C51, respectively.

*D*—H⋯*A*	*D*—H	H⋯*A*	*D*⋯*A*	*D*—H⋯*A*
C3—H3*A*⋯*Cg*2	0.97	2.87	3.677 (4)	141
C3—H3*B*⋯*Cg*3	0.97	2.75	3.562 (4)	141
C52—H52*A*⋯*Cg*1^i^	0.97	2.44	3.406 (7)	171
C52—H52*B*⋯*Cg*4^i^	0.97	2.62	3.434 (7)	141
C53—H53*A*⋯*Cg*4^ii^	0.97	2.88	3.818 (8)	162
C53—H53*B*⋯*Cg*1	0.97	2.63	3.585 (8)	169

Symmetry codes: (i) 
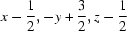
; (ii) 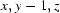.

**Fig. 1.**
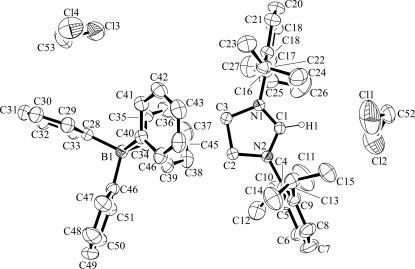
The asymmetric unit of the title compound, with atom labels and 30% probability displacement ellipsoids for non-H atoms.

## supplementary crystallographic information

### Comment

Imidazolium and imidazolidinium cations are the essential precursors to neutral free carbene compounds in which a divalent carbon with a six-electron valence shell is electronically stabilized by donating amino-substituents and sterically protected by substituted phenyl groups. The title compound is related to the previously prepared 1,3-bis(2,6-diisopropylphenyl)imidazolidinium chloride. As a result of the increased steric bulk associated with the tetraphenylborate anion the title compound is the first reported structure in which the imidazolidinium C-2 hydrogen atom is not within the sum of the Van der Waals radii of any atom in the anion or co-crystalized solvent. The title compound can be compared to previously published structures (Arduengo *et al.*, 1995, 1999; Fliedel *et al.*, 2007; Hagos *et al.*, 2008).

The asymmetric unit of the title compound is shown in Fig. 1. The crystal structure is stabilized by weak intermolecular C-H···π(arene) interactions.

### Experimental

1,3-*bis*(2,6-diisopropylphenyl)imidazolidinium tetraphenylborate dichloromethane disolvate was prepared by reacting 1.00 g (2.34 mol) of the corresponding imidazolidinum chloride (Arduengo *et al.*, 1999) with one equivalent of sodium tetraphenylborate (0.80 g, 2.34 mmol) in dichloromethane. After stirring overnight, the solution was filtered through diatomaceous earth and allowed to slowly evaporate yielding colorless block-like crystals of the title compound. The proton NMR matched that in the literature of the imidazolidinum chloride and is typical of a tetraphenylborate anion.

### Refinement

The H atoms were placed in geometrically idealized positions with C—H distances of 0.93Å (aromatic), 0.98Å (idealized tertiary), 0.97Å (idealized secondary) and 0.96Å (idealized methyl). H atoms were constrained to ride on the parent C atom with *U*_iso_(H) = 1.2U_eq_(C) for aromatic and secondary protons and *U*_iso_(H) = 1.5U_eq_(C) for the idealized methyl and tertiary protons.

In order to obtain satisfactory thermal parameters for the model, SIMU and DELU restraints were applied to carbon atoms C4>C51.

### Crystal data

**Table:**

C_27_H_39_N_2_^+^·C_24_H_20_B^−^·2CH_2_Cl_2_	*F*(000) = 1872
*M**_r_* = 880.66	*D*_x_ = 1.155 Mg m^−^^3^
Monoclinic, *C**c*	Melting point = 472–475 K
Hall symbol: C -2yc	Mo *K*α radiation, λ = 0.71073 Å
*a* = 21.4648 (14) Å	Cell parameters from 7600 reflections
*b* = 10.3964 (7) Å	θ = 2.2–25.8°
*c* = 22.7524 (15) Å	µ = 0.27 mm^−^^1^
β = 93.760 (1)°	*T* = 296 K
*V* = 5066.4 (6) Å^3^	Block, yellow
*Z* = 4	0.49 × 0.34 × 0.29 mm

### Data collection

**Table:**

Bruker APEXII CCD diffractometer	8396 independent reflections
Radiation source: fine-focus sealed tube	7118 reflections with *I* > 2σ(*I*)
graphite	*R*_int_ = 0.013
φ and ω scans	θ_max_ = 26.0°, θ_min_ = 2.2°
Absorption correction: multi-scan (*SADABS*; Bruker, 2010)	*h* = −26→26
*T*_min_ = 0.675, *T*_max_ = 0.746	*k* = −12→12
17851 measured reflections	*l* = −22→28

### Refinement

**Table:**

Refinement on *F*^2^	Secondary atom site location: difference Fourier map
Least-squares matrix: full	Hydrogen site location: inferred from neighbouring sites
*R*[*F*^2^ > 2σ(*F*^2^)] = 0.062	H-atom parameters constrained
*wR*(*F*^2^) = 0.186	*w* = 1/[σ^2^(*F*_o_^2^) + (0.0829*P*)^2^ + 1.5238*P*] where *P* = (*F*_o_^2^ + 2*F*_c_^2^)/3
*S* = 1.35	(Δ/σ)_max_ = 0.001
8396 reflections	Δρ_max_ = 0.41 e Å^−^^3^
549 parameters	Δρ_min_ = −0.44 e Å^−^^3^
892 restraints	Absolute structure: Flack (1983), 3456 Friedels
Primary atom site location: structure-invariant direct methods	Flack parameter: −0.11 (10)

### Special details

**Table:**

Geometry. All e.s.d.'s (except the e.s.d. in the dihedral angle between two l.s. planes) are estimated using the full covariance matrix. The cell e.s.d.'s are taken into account individually in the estimation of e.s.d.'s in distances, angles and torsion angles; correlations between e.s.d.'s in cell parameters are only used when they are defined by crystal symmetry. An approximate (isotropic) treatment of cell e.s.d.'s is used for estimating e.s.d.'s involving l.s. planes.
Refinement. Refinement of *F*^2^ against ALL reflections. The weighted *R*-factor *wR* and goodness of fit *S* are based on *F*^2^, conventional *R*-factors *R* are based on *F*, with *F* set to zero for negative *F*^2^. The threshold expression of *F*^2^ > σ(*F*^2^) is used only for calculating *R*-factors(gt) *etc*. and is not relevant to the choice of reflections for refinement. *R*-factors based on *F*^2^ are statistically about twice as large as those based on *F*, and *R*- factors based on ALL data will be even larger.

### Fractional atomic coordinates and isotropic or equivalent isotropic displacement parameters (Å^2^)

**Table:**

	*x*	*y*	*z*	*U*_iso_*/*U*_eq_	
C1	0.32237 (14)	0.8042 (3)	0.34114 (15)	0.0434 (7)	
H1	0.2942	0.8353	0.3118	0.052*	
C2	0.39497 (16)	0.8011 (3)	0.41785 (18)	0.0519 (9)	
H2A	0.3814	0.8215	0.4566	0.062*	
H2B	0.4396	0.8151	0.4177	0.062*	
C3	0.37791 (15)	0.6632 (3)	0.40044 (16)	0.0461 (8)	
H3A	0.4129	0.6188	0.3846	0.055*	
H3B	0.3640	0.6149	0.4336	0.055*	
C4	0.36517 (16)	1.0162 (3)	0.3702 (2)	0.0583 (6)	
C5	0.4123 (2)	1.0698 (4)	0.3383 (2)	0.0696 (7)	
C6	0.4195 (2)	1.2020 (4)	0.3413 (3)	0.0763 (7)	
H6	0.4503	1.2417	0.3207	0.092*	
C7	0.3818 (2)	1.2746 (5)	0.3742 (3)	0.0799 (8)	
H7	0.3876	1.3632	0.3757	0.096*	
C8	0.3361 (2)	1.2214 (4)	0.4047 (3)	0.0740 (7)	
H8	0.3109	1.2739	0.4262	0.089*	
C9	0.32666 (18)	1.0890 (4)	0.4041 (2)	0.0657 (7)	
C10	0.4535 (2)	0.9890 (5)	0.3013 (3)	0.0765 (7)	
H10	0.4493	0.8989	0.3130	0.092*	
C11	0.4323 (3)	1.0003 (8)	0.2363 (3)	0.1121 (14)	
H11A	0.4347	1.0885	0.2242	0.168*	
H11B	0.3899	0.9708	0.2303	0.168*	
H11C	0.4588	0.9486	0.2134	0.168*	
C12	0.5217 (2)	1.0247 (6)	0.3085 (3)	0.0886 (12)	
H12A	0.5279	1.1069	0.2906	0.133*	
H12B	0.5460	0.9609	0.2899	0.133*	
H12C	0.5345	1.0291	0.3497	0.133*	
C13	0.27496 (19)	1.0312 (4)	0.4373 (2)	0.0700 (7)	
H13	0.2771	0.9376	0.4325	0.084*	
C14	0.2814 (3)	1.0592 (6)	0.5022 (3)	0.0930 (11)	
H14A	0.3187	1.0193	0.5192	0.139*	
H14B	0.2459	1.0257	0.5206	0.139*	
H14C	0.2839	1.1505	0.5083	0.139*	
C15	0.2115 (2)	1.0757 (5)	0.4093 (3)	0.0831 (11)	
H15A	0.2056	1.1651	0.4178	0.125*	
H15B	0.1788	1.0262	0.4251	0.125*	
H15C	0.2106	1.0635	0.3674	0.125*	
C16	0.29202 (19)	0.5791 (4)	0.3253 (2)	0.0599 (6)	
C17	0.3132 (2)	0.5293 (4)	0.2739 (2)	0.0692 (7)	
C18	0.2803 (2)	0.4256 (4)	0.2483 (2)	0.0752 (8)	
H18	0.2931	0.3896	0.2136	0.090*	
C19	0.2297 (2)	0.3767 (4)	0.2736 (2)	0.0766 (8)	
H19	0.2086	0.3074	0.2558	0.092*	
C20	0.2087 (2)	0.4267 (4)	0.3249 (2)	0.0715 (7)	
H20	0.1740	0.3912	0.3411	0.086*	
C21	0.23988 (19)	0.5310 (4)	0.3526 (2)	0.0623 (6)	
C22	0.2165 (2)	0.5901 (4)	0.4072 (2)	0.0673 (7)	
H22	0.2524	0.6303	0.4287	0.081*	
C23	0.1891 (2)	0.4947 (5)	0.4486 (3)	0.0830 (10)	
H23A	0.1542	0.4517	0.4286	0.125*	
H23B	0.1753	0.5393	0.4823	0.125*	
H23C	0.2203	0.4325	0.4610	0.125*	
C24	0.1694 (2)	0.6984 (5)	0.3905 (3)	0.0860 (11)	
H24A	0.1898	0.7650	0.3698	0.129*	
H24B	0.1536	0.7334	0.4256	0.129*	
H24C	0.1354	0.6642	0.3657	0.129*	
C25	0.3687 (3)	0.5847 (5)	0.2449 (2)	0.0779 (8)	
H25	0.3918	0.6392	0.2740	0.094*	
C26	0.4138 (3)	0.4820 (6)	0.2250 (3)	0.0993 (13)	
H26A	0.4349	0.4427	0.2588	0.149*	
H26B	0.4438	0.5211	0.2011	0.149*	
H26C	0.3908	0.4176	0.2024	0.149*	
C27	0.3473 (4)	0.6694 (6)	0.1931 (3)	0.1047 (13)	
H27A	0.3293	0.6168	0.1618	0.157*	
H27B	0.3825	0.7154	0.1797	0.157*	
H27C	0.3167	0.7295	0.2052	0.157*	
C28	0.56734 (17)	0.4344 (3)	0.57017 (18)	0.0519 (7)	
C29	0.5472 (3)	0.3788 (4)	0.6212 (2)	0.0755 (10)	
H29	0.5109	0.4098	0.6364	0.091*	
C30	0.5791 (3)	0.2785 (5)	0.6505 (3)	0.0988 (14)	
H30	0.5649	0.2456	0.6852	0.119*	
C31	0.6317 (3)	0.2287 (5)	0.6279 (3)	0.1040 (15)	
H31	0.6526	0.1605	0.6467	0.125*	
C32	0.6528 (3)	0.2785 (5)	0.5786 (3)	0.0925 (13)	
H32	0.6886	0.2449	0.5634	0.111*	
C33	0.62107 (19)	0.3813 (4)	0.5497 (2)	0.0685 (9)	
H33	0.6367	0.4149	0.5158	0.082*	
C34	0.54183 (14)	0.5733 (3)	0.46984 (17)	0.0475 (6)	
C35	0.53663 (18)	0.4641 (4)	0.4336 (2)	0.0611 (8)	
H35	0.5308	0.3845	0.4511	0.073*	
C36	0.5398 (2)	0.4689 (5)	0.3730 (2)	0.0739 (9)	
H36	0.5363	0.3936	0.3510	0.089*	
C37	0.5479 (2)	0.5841 (6)	0.3457 (2)	0.0784 (10)	
H37	0.5505	0.5879	0.3051	0.094*	
C38	0.55225 (18)	0.6931 (5)	0.3788 (2)	0.0702 (9)	
H38	0.5575	0.7720	0.3605	0.084*	
C39	0.54900 (15)	0.6885 (4)	0.43912 (19)	0.0570 (7)	
H39	0.5517	0.7650	0.4603	0.068*	
C40	0.45929 (16)	0.5707 (3)	0.54864 (17)	0.0503 (7)	
C41	0.42234 (18)	0.4617 (4)	0.5400 (2)	0.0597 (8)	
H41	0.4411	0.3839	0.5313	0.072*	
C42	0.35757 (19)	0.4663 (5)	0.5439 (2)	0.0717 (10)	
H42	0.3345	0.3909	0.5388	0.086*	
C43	0.3276 (2)	0.5772 (5)	0.5550 (2)	0.0760 (10)	
H43	0.2844	0.5794	0.5566	0.091*	
C44	0.3623 (2)	0.6847 (5)	0.5637 (2)	0.0743 (10)	
H44	0.3428	0.7615	0.5725	0.089*	
C45	0.42724 (18)	0.6826 (4)	0.5598 (2)	0.0631 (9)	
H45	0.4495	0.7590	0.5648	0.076*	
C46	0.57412 (16)	0.6805 (3)	0.57753 (18)	0.0528 (7)	
C47	0.5579 (2)	0.7240 (5)	0.6326 (2)	0.0745 (10)	
H47	0.5218	0.6929	0.6481	0.089*	
C48	0.5949 (3)	0.8135 (6)	0.6650 (3)	0.0917 (13)	
H48	0.5823	0.8418	0.7011	0.110*	
C49	0.6482 (3)	0.8594 (5)	0.6452 (3)	0.0913 (13)	
H49	0.6722	0.9183	0.6674	0.110*	
C50	0.6665 (2)	0.8187 (4)	0.5921 (3)	0.0786 (11)	
H50	0.7034	0.8493	0.5780	0.094*	
C51	0.62988 (18)	0.7313 (4)	0.5591 (2)	0.0615 (8)	
H51	0.6431	0.7052	0.5229	0.074*	
C52	0.1967 (3)	0.9707 (6)	0.1819 (3)	0.109 (2)	
H52B	0.1940	0.8877	0.1624	0.130*	
H52A	0.1664	1.0279	0.1622	0.130*	
C53	0.5164 (3)	0.0513 (8)	0.5452 (3)	0.115 (2)	
H53B	0.5442	0.1185	0.5607	0.138*	
H53A	0.5363	−0.0309	0.5541	0.138*	
B1	0.53552 (17)	0.5659 (4)	0.54107 (19)	0.0459 (8)	
Cl1	0.18097 (19)	0.9538 (4)	0.25374 (12)	0.2196 (19)	
Cl2	0.26822 (14)	1.0308 (4)	0.1782 (2)	0.230 (2)	
Cl3	0.44650 (12)	0.0595 (2)	0.57959 (13)	0.1433 (8)	
Cl4	0.50522 (12)	0.0685 (2)	0.47076 (10)	0.1297 (7)	
N1	0.32668 (13)	0.6829 (2)	0.35495 (13)	0.0462 (6)	
N2	0.36081 (11)	0.8780 (2)	0.37186 (13)	0.0442 (6)	

### Atomic displacement parameters (Å^2^)

**Table:**

	*U*^11^	*U*^22^	*U*^33^	*U*^12^	*U*^13^	*U*^23^
C1	0.0402 (14)	0.0456 (16)	0.0443 (19)	0.0018 (12)	0.0016 (13)	0.0055 (14)
C2	0.0501 (17)	0.0423 (16)	0.061 (2)	0.0011 (13)	−0.0120 (16)	−0.0010 (15)
C3	0.0454 (16)	0.0414 (16)	0.051 (2)	0.0015 (12)	−0.0001 (14)	0.0040 (14)
C4	0.0478 (12)	0.0457 (11)	0.0807 (18)	−0.0015 (10)	−0.0008 (10)	0.0059 (12)
C5	0.0597 (13)	0.0571 (11)	0.0923 (18)	−0.0076 (11)	0.0079 (12)	0.0098 (13)
C6	0.0705 (15)	0.0577 (11)	0.101 (2)	−0.0091 (12)	0.0120 (13)	0.0141 (14)
C7	0.0773 (16)	0.0564 (13)	0.106 (2)	−0.0047 (11)	0.0063 (14)	0.0069 (13)
C8	0.0679 (15)	0.0517 (11)	0.103 (2)	0.0055 (11)	0.0079 (13)	0.0004 (14)
C9	0.0553 (12)	0.0508 (11)	0.0909 (18)	0.0048 (10)	0.0028 (11)	0.0037 (13)
C10	0.0697 (14)	0.0664 (15)	0.0950 (18)	−0.0061 (13)	0.0179 (12)	0.0091 (15)
C11	0.107 (3)	0.132 (3)	0.0970 (18)	0.001 (3)	0.004 (2)	−0.012 (3)
C12	0.0680 (14)	0.095 (3)	0.105 (3)	−0.0053 (18)	0.0219 (16)	0.015 (2)
C13	0.0604 (13)	0.0580 (15)	0.0924 (17)	0.0066 (12)	0.0116 (11)	0.0007 (15)
C14	0.077 (2)	0.112 (3)	0.0894 (16)	0.001 (2)	0.0054 (15)	0.004 (2)
C15	0.0565 (13)	0.096 (3)	0.097 (3)	0.0003 (18)	0.0058 (17)	0.001 (2)
C16	0.0669 (14)	0.0528 (14)	0.0585 (14)	−0.0079 (11)	−0.0077 (10)	−0.0048 (11)
C17	0.0814 (15)	0.0645 (15)	0.0605 (15)	−0.0071 (11)	−0.0034 (11)	−0.0080 (11)
C18	0.0887 (17)	0.0692 (16)	0.0664 (17)	−0.0067 (12)	−0.0041 (13)	−0.0160 (13)
C19	0.0867 (16)	0.0668 (16)	0.0742 (16)	−0.0129 (13)	−0.0099 (13)	−0.0131 (12)
C20	0.0742 (16)	0.0645 (15)	0.0743 (17)	−0.0164 (12)	−0.0060 (13)	−0.0075 (12)
C21	0.0648 (14)	0.0550 (14)	0.0656 (14)	−0.0070 (10)	−0.0069 (10)	−0.0036 (11)
C22	0.0648 (15)	0.0627 (15)	0.0740 (16)	−0.0080 (11)	0.0003 (12)	−0.0079 (11)
C23	0.083 (2)	0.081 (2)	0.086 (2)	−0.0095 (19)	0.0163 (19)	−0.0005 (18)
C24	0.081 (2)	0.076 (2)	0.101 (3)	0.0090 (16)	0.007 (2)	−0.0066 (18)
C25	0.0927 (17)	0.0788 (17)	0.0623 (16)	−0.0117 (12)	0.0057 (12)	−0.0089 (14)
C26	0.107 (3)	0.103 (3)	0.091 (3)	0.005 (2)	0.026 (2)	0.001 (2)
C27	0.138 (3)	0.094 (3)	0.083 (3)	0.001 (2)	0.019 (2)	0.015 (2)
C28	0.0546 (15)	0.0444 (16)	0.0549 (19)	−0.0039 (12)	−0.0088 (14)	−0.0031 (12)
C29	0.097 (3)	0.067 (2)	0.061 (2)	−0.0109 (18)	−0.0046 (17)	0.0102 (18)
C30	0.139 (4)	0.073 (3)	0.080 (3)	−0.017 (2)	−0.029 (2)	0.024 (2)
C31	0.133 (4)	0.059 (2)	0.111 (3)	0.007 (2)	−0.059 (3)	0.005 (2)
C32	0.092 (3)	0.070 (2)	0.110 (3)	0.026 (2)	−0.037 (2)	−0.016 (2)
C33	0.0610 (19)	0.060 (2)	0.082 (3)	0.0117 (15)	−0.0103 (15)	−0.0099 (17)
C34	0.0359 (14)	0.0574 (14)	0.0495 (17)	0.0049 (13)	0.0041 (13)	−0.0002 (10)
C35	0.061 (2)	0.0652 (16)	0.0569 (16)	−0.0043 (17)	0.0035 (18)	−0.0077 (14)
C36	0.066 (2)	0.100 (2)	0.0559 (16)	−0.005 (2)	0.0018 (19)	−0.0176 (17)
C37	0.060 (2)	0.125 (2)	0.050 (2)	0.002 (2)	0.0022 (19)	0.0067 (14)
C38	0.0514 (18)	0.093 (2)	0.0668 (17)	0.0095 (19)	0.0094 (18)	0.0248 (16)
C39	0.0444 (16)	0.0613 (14)	0.0660 (17)	0.0105 (14)	0.0084 (16)	0.0087 (14)
C40	0.0483 (14)	0.0616 (15)	0.0416 (19)	0.0010 (10)	0.0076 (14)	0.0062 (15)
C41	0.0538 (14)	0.0676 (16)	0.058 (2)	−0.0038 (13)	0.0069 (16)	0.0028 (18)
C42	0.0538 (14)	0.095 (2)	0.066 (3)	−0.0157 (15)	0.0072 (18)	0.005 (2)
C43	0.0481 (17)	0.119 (2)	0.063 (3)	0.0053 (13)	0.0142 (18)	0.013 (2)
C44	0.0615 (15)	0.093 (2)	0.070 (3)	0.0218 (15)	0.015 (2)	0.010 (2)
C45	0.0595 (14)	0.0641 (16)	0.067 (3)	0.0081 (13)	0.0115 (18)	0.0081 (18)
C46	0.0545 (15)	0.0440 (16)	0.0599 (19)	0.0040 (12)	0.0037 (14)	−0.0047 (14)
C47	0.072 (2)	0.083 (3)	0.070 (2)	0.0053 (18)	0.0111 (17)	−0.020 (2)
C48	0.098 (3)	0.096 (3)	0.080 (3)	0.012 (2)	−0.005 (2)	−0.038 (2)
C49	0.102 (3)	0.064 (2)	0.104 (3)	−0.005 (2)	−0.022 (2)	−0.023 (2)
C50	0.072 (2)	0.066 (2)	0.096 (3)	−0.0150 (17)	−0.0113 (19)	0.002 (2)
C51	0.0600 (17)	0.0571 (19)	0.067 (2)	−0.0048 (14)	0.0018 (15)	0.0026 (16)
C52	0.124 (5)	0.100 (4)	0.094 (5)	0.022 (3)	−0.042 (4)	−0.013 (3)
C53	0.087 (4)	0.142 (6)	0.111 (6)	−0.021 (3)	−0.031 (4)	0.005 (4)
B1	0.0414 (17)	0.0488 (19)	0.048 (2)	0.0026 (14)	0.0048 (15)	0.0023 (16)
Cl1	0.261 (4)	0.315 (5)	0.0782 (15)	0.095 (3)	−0.0274 (19)	−0.015 (2)
Cl2	0.1225 (17)	0.213 (3)	0.342 (6)	0.0025 (19)	−0.077 (3)	0.004 (3)
Cl3	0.1502 (17)	0.1418 (17)	0.143 (2)	−0.0151 (12)	0.0490 (14)	−0.0088 (13)
Cl4	0.1669 (18)	0.1260 (14)	0.0946 (13)	−0.0198 (12)	−0.0045 (12)	−0.0084 (10)
N1	0.0494 (14)	0.0403 (13)	0.0480 (17)	−0.0017 (11)	−0.0037 (12)	0.0004 (12)
N2	0.0376 (12)	0.0412 (13)	0.0534 (18)	−0.0028 (10)	−0.0004 (11)	0.0046 (12)

### Geometric parameters (Å, °)

**Table:**

C1—N2	1.297 (4)	C25—H25	0.9800
C1—N1	1.301 (4)	C26—H26A	0.9600
C1—H1	0.9300	C26—H26B	0.9600
C2—N2	1.474 (4)	C26—H26C	0.9600
C2—C3	1.526 (5)	C27—H27A	0.9600
C2—H2A	0.9700	C27—H27B	0.9600
C2—H2B	0.9700	C27—H27C	0.9600
C3—N1	1.474 (4)	C28—C29	1.390 (6)
C3—H3A	0.9700	C28—C33	1.386 (6)
C3—H3B	0.9700	C28—B1	1.648 (5)
C4—C9	1.391 (6)	C29—C30	1.393 (7)
C4—C5	1.398 (6)	C29—H29	0.9300
C4—N2	1.441 (5)	C30—C31	1.372 (10)
C5—C6	1.385 (6)	C30—H30	0.9300
C5—C10	1.514 (7)	C31—C32	1.340 (10)
C6—C7	1.366 (7)	C31—H31	0.9300
C6—H6	0.9300	C32—C33	1.407 (7)
C7—C8	1.357 (7)	C32—H32	0.9300
C7—H7	0.9300	C33—H33	0.9300
C8—C9	1.392 (6)	C34—C39	1.400 (5)
C8—H8	0.9300	C34—C35	1.403 (5)
C9—C13	1.507 (7)	C34—B1	1.637 (6)
C10—C12	1.509 (6)	C35—C36	1.384 (7)
C10—C11	1.523 (9)	C35—H35	0.9300
C10—H10	0.9800	C36—C37	1.367 (7)
C11—H11A	0.9600	C36—H36	0.9300
C11—H11B	0.9600	C37—C38	1.362 (7)
C11—H11C	0.9600	C37—H37	0.9300
C12—H12A	0.9600	C38—C39	1.378 (6)
C12—H12B	0.9600	C38—H38	0.9300
C12—H12C	0.9600	C39—H39	0.9300
C13—C14	1.503 (8)	C40—C45	1.383 (5)
C13—C15	1.536 (6)	C40—C41	1.390 (5)
C13—H13	0.9800	C40—B1	1.657 (5)
C14—H14A	0.9600	C41—C42	1.400 (6)
C14—H14B	0.9600	C41—H41	0.9300
C14—H14C	0.9600	C42—C43	1.352 (7)
C15—H15A	0.9600	C42—H42	0.9300
C15—H15B	0.9600	C43—C44	1.350 (7)
C15—H15C	0.9600	C43—H43	0.9300
C16—C17	1.381 (7)	C44—C45	1.403 (6)
C16—C21	1.407 (7)	C44—H44	0.9300
C16—N1	1.452 (4)	C45—H45	0.9300
C17—C18	1.396 (6)	C46—C47	1.397 (6)
C17—C25	1.514 (7)	C46—C51	1.397 (6)
C18—C19	1.361 (8)	C46—B1	1.645 (5)
C18—H18	0.9300	C47—C48	1.401 (7)
C19—C20	1.381 (7)	C47—H47	0.9300
C19—H19	0.9300	C48—C49	1.344 (9)
C20—C21	1.401 (6)	C48—H48	0.9300
C20—H20	0.9300	C49—C50	1.362 (8)
C21—C22	1.502 (7)	C49—H49	0.9300
C22—C23	1.513 (7)	C50—C51	1.389 (6)
C22—C24	1.544 (6)	C50—H50	0.9300
C22—H22	0.9800	C51—H51	0.9300
C23—H23A	0.9600	C52—Cl2	1.665 (8)
C23—H23B	0.9600	C52—Cl1	1.699 (9)
C23—H23C	0.9600	C52—H52B	0.9700
C24—H24A	0.9600	C52—H52A	0.9700
C24—H24B	0.9600	C53—Cl4	1.705 (8)
C24—H24C	0.9600	C53—Cl3	1.739 (8)
C25—C27	1.518 (8)	C53—H53B	0.9700
C25—C26	1.530 (8)	C53—H53A	0.9700
			
N2—C1—N1	114.2 (3)	C26—C25—H25	107.6
N2—C1—H1	122.9	C25—C26—H26A	109.5
N1—C1—H1	122.9	C25—C26—H26B	109.5
N2—C2—C3	103.1 (3)	H26A—C26—H26B	109.5
N2—C2—H2A	111.2	C25—C26—H26C	109.5
C3—C2—H2A	111.2	H26A—C26—H26C	109.5
N2—C2—H2B	111.2	H26B—C26—H26C	109.5
C3—C2—H2B	111.2	C25—C27—H27A	109.5
H2A—C2—H2B	109.1	C25—C27—H27B	109.5
N1—C3—C2	101.9 (2)	H27A—C27—H27B	109.5
N1—C3—H3A	111.4	C25—C27—H27C	109.5
C2—C3—H3A	111.4	H27A—C27—H27C	109.5
N1—C3—H3B	111.4	H27B—C27—H27C	109.5
C2—C3—H3B	111.4	C29—C28—C33	115.2 (4)
H3A—C3—H3B	109.2	C29—C28—B1	122.5 (4)
C9—C4—C5	123.2 (4)	C33—C28—B1	121.8 (4)
C9—C4—N2	119.0 (3)	C28—C29—C30	122.7 (5)
C5—C4—N2	117.5 (4)	C28—C29—H29	118.7
C6—C5—C4	116.9 (4)	C30—C29—H29	118.7
C6—C5—C10	120.6 (4)	C31—C30—C29	119.6 (6)
C4—C5—C10	122.4 (4)	C31—C30—H30	120.2
C7—C6—C5	120.4 (4)	C29—C30—H30	120.2
C7—C6—H6	119.8	C32—C31—C30	120.0 (5)
C5—C6—H6	119.8	C32—C31—H31	120.0
C8—C7—C6	122.0 (4)	C30—C31—H31	120.0
C8—C7—H7	119.0	C31—C32—C33	120.3 (6)
C6—C7—H7	119.0	C31—C32—H32	119.9
C7—C8—C9	120.5 (4)	C33—C32—H32	119.9
C7—C8—H8	119.7	C28—C33—C32	122.3 (5)
C9—C8—H8	119.7	C28—C33—H33	118.9
C8—C9—C4	116.8 (4)	C32—C33—H33	118.9
C8—C9—C13	120.1 (4)	C39—C34—C35	113.9 (4)
C4—C9—C13	123.0 (3)	C39—C34—B1	123.6 (3)
C12—C10—C5	113.6 (4)	C35—C34—B1	122.4 (3)
C12—C10—C11	108.3 (5)	C36—C35—C34	123.3 (4)
C5—C10—C11	110.5 (5)	C36—C35—H35	118.4
C12—C10—H10	108.1	C34—C35—H35	118.4
C5—C10—H10	108.1	C37—C36—C35	120.1 (5)
C11—C10—H10	108.1	C37—C36—H36	120.0
C10—C11—H11A	109.5	C35—C36—H36	120.0
C10—C11—H11B	109.5	C36—C37—C38	118.9 (5)
H11A—C11—H11B	109.5	C36—C37—H37	120.6
C10—C11—H11C	109.5	C38—C37—H37	120.6
H11A—C11—H11C	109.5	C37—C38—C39	121.1 (4)
H11B—C11—H11C	109.5	C37—C38—H38	119.5
C10—C12—H12A	109.5	C39—C38—H38	119.5
C10—C12—H12B	109.5	C38—C39—C34	122.8 (4)
H12A—C12—H12B	109.5	C38—C39—H39	118.6
C10—C12—H12C	109.5	C34—C39—H39	118.6
H12A—C12—H12C	109.5	C45—C40—C41	115.2 (3)
H12B—C12—H12C	109.5	C45—C40—B1	123.4 (3)
C14—C13—C9	113.0 (4)	C41—C40—B1	121.2 (3)
C14—C13—C15	111.9 (4)	C42—C41—C40	121.5 (4)
C9—C13—C15	109.5 (4)	C42—C41—H41	119.2
C14—C13—H13	107.4	C40—C41—H41	119.2
C9—C13—H13	107.4	C43—C42—C41	121.8 (4)
C15—C13—H13	107.4	C43—C42—H42	119.1
C13—C14—H14A	109.5	C41—C42—H42	119.1
C13—C14—H14B	109.5	C44—C43—C42	117.9 (4)
H14A—C14—H14B	109.5	C44—C43—H43	121.0
C13—C14—H14C	109.5	C42—C43—H43	121.0
H14A—C14—H14C	109.5	C43—C44—C45	121.3 (4)
H14B—C14—H14C	109.5	C43—C44—H44	119.4
C13—C15—H15A	109.5	C45—C44—H44	119.4
C13—C15—H15B	109.5	C40—C45—C44	122.2 (4)
H15A—C15—H15B	109.5	C40—C45—H45	118.9
C13—C15—H15C	109.5	C44—C45—H45	118.9
H15A—C15—H15C	109.5	C47—C46—C51	114.5 (4)
H15B—C15—H15C	109.5	C47—C46—B1	122.7 (4)
C17—C16—C21	123.7 (4)	C51—C46—B1	122.4 (4)
C17—C16—N1	119.0 (4)	C48—C47—C46	121.3 (5)
C21—C16—N1	117.2 (4)	C48—C47—H47	119.3
C16—C17—C18	117.3 (5)	C46—C47—H47	119.3
C16—C17—C25	122.6 (4)	C49—C48—C47	121.6 (5)
C18—C17—C25	120.1 (5)	C49—C48—H48	119.2
C19—C18—C17	120.4 (5)	C47—C48—H48	119.2
C19—C18—H18	119.8	C50—C49—C48	119.3 (5)
C17—C18—H18	119.8	C50—C49—H49	120.3
C18—C19—C20	122.2 (4)	C48—C49—H49	120.3
C18—C19—H19	118.9	C49—C50—C51	119.6 (5)
C20—C19—H19	118.9	C49—C50—H50	120.2
C19—C20—C21	119.9 (5)	C51—C50—H50	120.2
C19—C20—H20	120.1	C50—C51—C46	123.5 (5)
C21—C20—H20	120.1	C50—C51—H51	118.3
C20—C21—C16	116.5 (4)	C46—C51—H51	118.3
C20—C21—C22	120.8 (4)	Cl2—C52—Cl1	109.2 (4)
C16—C21—C22	122.6 (3)	Cl2—C52—H52B	109.8
C21—C22—C23	114.3 (4)	Cl1—C52—H52B	109.8
C21—C22—C24	110.2 (4)	Cl2—C52—H52A	109.8
C23—C22—C24	110.9 (4)	Cl1—C52—H52A	109.8
C21—C22—H22	107.1	H52B—C52—H52A	108.3
C23—C22—H22	107.1	Cl4—C53—Cl3	111.8 (3)
C24—C22—H22	107.1	Cl4—C53—H53B	109.3
C22—C23—H23A	109.5	Cl3—C53—H53B	109.3
C22—C23—H23B	109.5	Cl4—C53—H53A	109.3
H23A—C23—H23B	109.5	Cl3—C53—H53A	109.3
C22—C23—H23C	109.5	H53B—C53—H53A	107.9
H23A—C23—H23C	109.5	C46—B1—C34	113.2 (3)
H23B—C23—H23C	109.5	C46—B1—C28	102.5 (3)
C22—C24—H24A	109.5	C34—B1—C28	112.2 (3)
C22—C24—H24B	109.5	C46—B1—C40	113.2 (3)
H24A—C24—H24B	109.5	C34—B1—C40	104.4 (3)
C22—C24—H24C	109.5	C28—B1—C40	111.7 (3)
H24A—C24—H24C	109.5	C1—N1—C16	125.6 (3)
H24B—C24—H24C	109.5	C1—N1—C3	110.0 (3)
C17—C25—C27	110.6 (5)	C16—N1—C3	123.9 (3)
C17—C25—C26	113.3 (4)	C1—N2—C4	128.0 (3)
C27—C25—C26	109.8 (5)	C1—N2—C2	109.3 (2)
C17—C25—H25	107.6	C4—N2—C2	122.0 (3)
C27—C25—H25	107.6		

### Hydrogen-bond geometry (Å, °)

**Table:**

Cg1, Cg2, Cg3 and Cg4 are the centroids defined by the ring atoms C28–C33, C34–C39, C40–C45 and C46–C51, respectively.

**Table:**

*D*—H···*A*	*D*—H	H···*A*	*D*···*A*	*D*—H···*A*
C3—H3A···Cg2	0.97	2.87	3.677 (4)	141
C3—H3B···Cg3	0.97	2.75	3.562 (4)	141
C52—H52A···Cg1^i^	0.97	2.44	3.406 (7)	171
C52—H52B···Cg4^i^	0.97	2.62	3.434 (7)	141
C53—H53A···Cg4^ii^	0.97	2.88	3.818 (8)	162
C53—H53B···Cg1	0.97	2.63	3.585 (8)	169

Symmetry codes: (i) *x*−1/2, −*y*+3/2, *z*−1/2; (ii) *x*, *y*−1, *z*.
